# Supplementary material for: SENP2 restrains the generation of pathogenic Th17 cells in mouse models of colitis
Source: Commun Biol. 2023 Jun 10;6:629. doi: 10.1038/s42003-023-05009-4 (PMC10257679; doi:10.1038/s42003-023-05009-4)
Supplement: Supplementary file 2 — Supplementary Information [file 42003_2023_5009_MOESM2_ESM.pdf]

Supplementary information

SENP2 restrains the generation of pathogenic Th17 cells in mouse models of colitis disease

Tsan-Tzu Yang, Ming-Feng Chiang, Che-Chang Chang, Shii-Yi Yang, Shih-Wen Huang, Nan-Shih Liao, Hsiu-Ming Shih, Wei Hsu and Kuo-I Lin*

Corresponding author: Kuo-I Lin
Email: kuoilin@gate.sinica.edu.tw

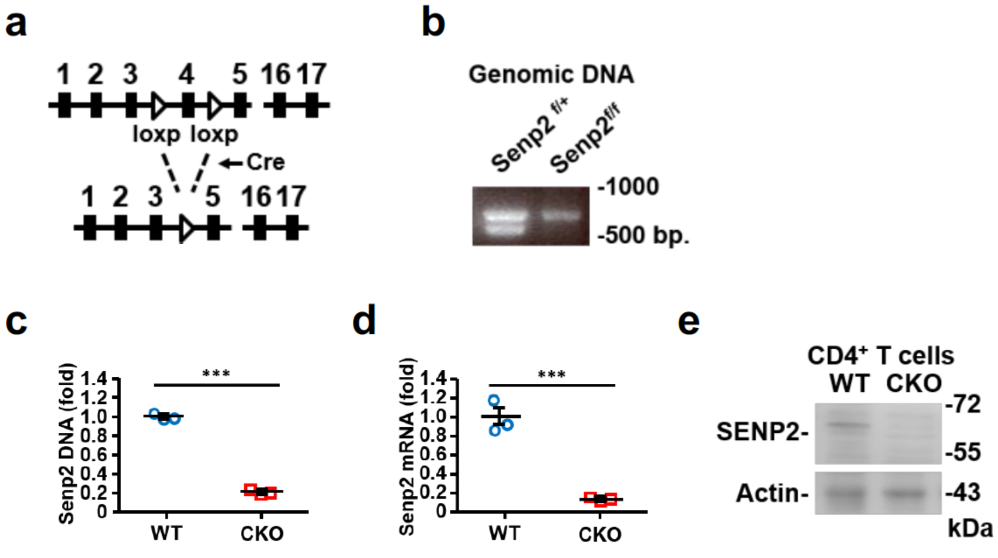

Supplemental Figure 1. Generation of T-cell-specific *Senp2* knockout mice.
(a) Strategy of generating *Senp2*-deficient (CKO) mice by inserting two floxed alleles (denoted by empty triangles) surrounding *Senp2* exon 4. In the presence of Cre, exon 4 of *Senp2* is deleted. (b–e) *Senp2* is effectively deleted in pan-T cells of CKO mice. The genomic DNA (b, c), RNA (d), and protein (e) of splenic pan-T cells were extracted from CKO and littermate wild-type (WT) mice, and subjected to genomic PCR (b), qPCR (c), RT-qPCR (d), and immunoblotting (e), respectively, to determine the efficiency of deletion of *Senp2*. Results in c and d are mean ± SD. n=3. Statistical analysis was done by Student's t test. ***$p < 0.001$.

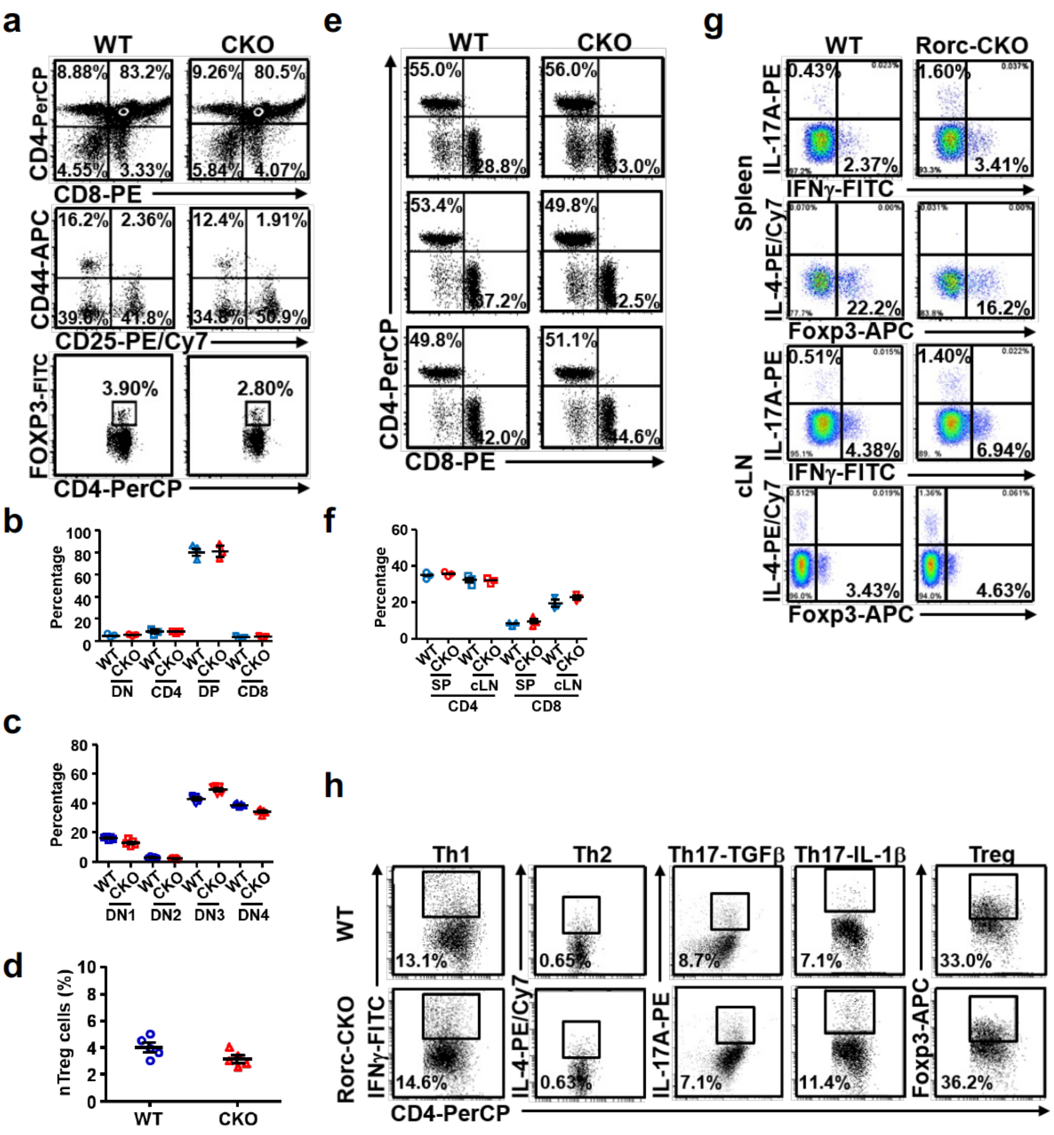

Supplemental Figure 2. Normal T-cell development but altered T-cell subset in mice lacking *Senp2* in T cells.

(a–d) FACS showing the frequency of developing T-cell thymocytes (top), double-negative (DN) T-cell thymocyte subsets (middle), and nTreg (bottom) cells in the thymus of WT and CKO mice (a). Statistical analysis of developing thymocytes is shown in (b–d). (e and f) FACS showing the frequency of $CD4^+$ and $CD8^+$ T cells in the spleen (top), cervical lymph node (cLN) (middle), and mesenteric lymph node (MLN) (bottom) of WT and CKO mice (e), and the statistical analysis of $CD4^+$ and $CD8^+$ T-cell frequency in SP and cLN is shown in f. (g) FACS showing the frequency of $IFN\gamma^+$- and $IL\text{-}17^+\text{-}CD4^+$ T cells in the spleen and cLN of WT and Rorc-CKO mice. (h) FACS showing the frequency of Th subsets by differentiating naïve splenic $CD4^+$ T cells from WT and Rorc-CKO mice for 5 days. Results in b, c, d, and f are mean ± SD. n= 3 in b and f, 5 in c and d. Statistical analysis was done by Student’s t test. Each symbol represents one animal.

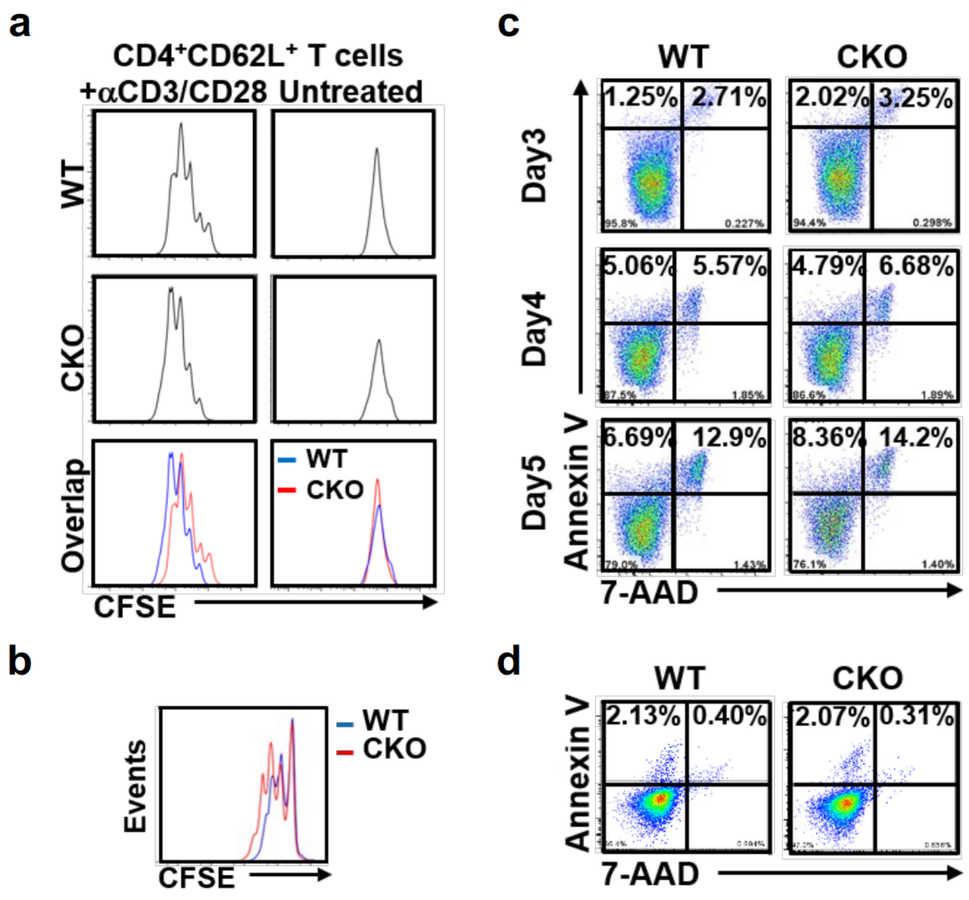

Supplemental Figure 3. Cell proliferation and apoptosis were not affected by deletion of *Senp2* in T cells.

(a) FACS analysis showing the cell proliferation as analyzed by CFSE staining. Naïve $CD4^+$ T cells were isolated from the spleen of WT and CKO mice, labeled with CFSE, and stimulated with anti-CD3 (5 μg/ml) + anti-CD28 (2 μg/ml) for 3 days. The untreated group was not treated with anti-CD3 + anti-CD28. FACS was performed on day 3. (b) FACS analysis showing the frequency of Annexin V^+ and/or 7-AAD^+ cells on the indicated days of anti-CD3 + anti-CD28 stimulated naïve $CD4^+$ T cells isolated from the spleen of WT and CKO mice. (c) FACS analysis showing cell proliferation in the pathogenic Th17 culture. Naïve $CD4^+$ T cells were isolated from the spleen of WT and CKO mice, labeled with CFSE, and cultured in polarizing conditions favoring pathogenic Th17 cells. FACS was analyzed on day 3. (d) FACS analysis showing the frequency of Annexin V^+ and/or 7-AAD^+ cells on day 3 of polarizing pathogenic Th17 cell culture derived from WT and CKO mice.

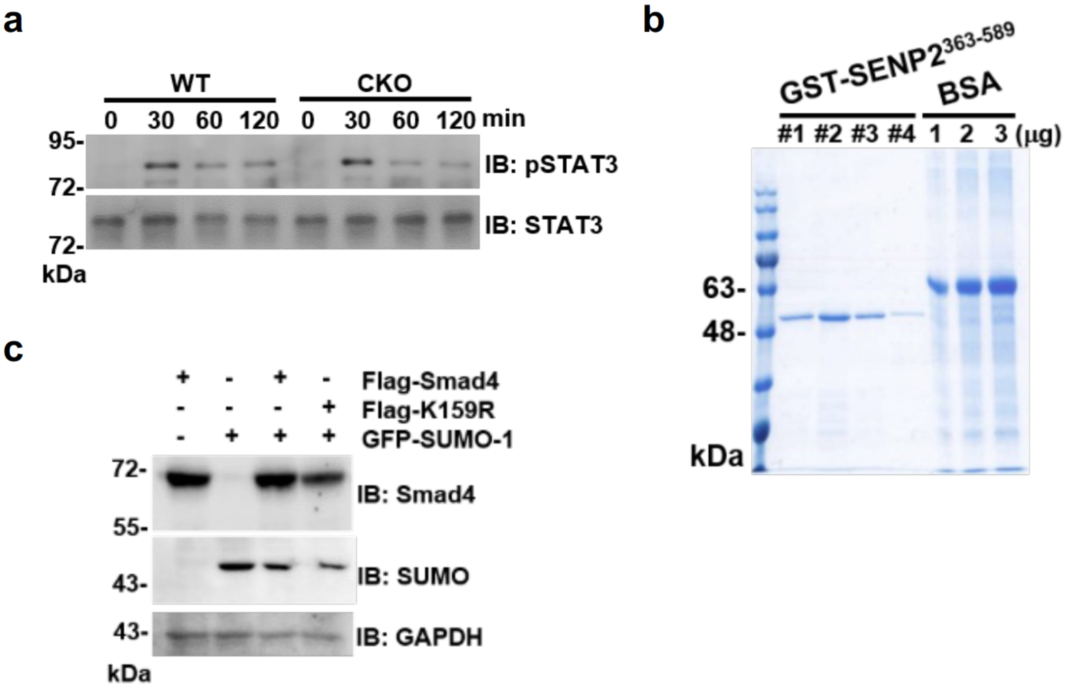

Supplemental Figure 4. Smad4 is modified by SUMO-1 at K159.

(a) Immunoblotting (IB) showing the levels of phospho-STAT3 at indicated time points of pathogenic Th17 culture derived from splenic $CD4^+$ T cells of WT and CKO mice. (b) Coomassie Brilliant Blue staining detected the purified recombinant GST-SENP2$^{363\text{-}589}$ fusion protein segment in various fractions. (c) IB showed the expression of exogenous Smad4 wild-type (WT) or Smad4 K159R, and exogenous GFP-SUMO-1 in EL4 cells on day 2 after transduction with indicated expression vectors. GAPDH was used for internal control blotting.

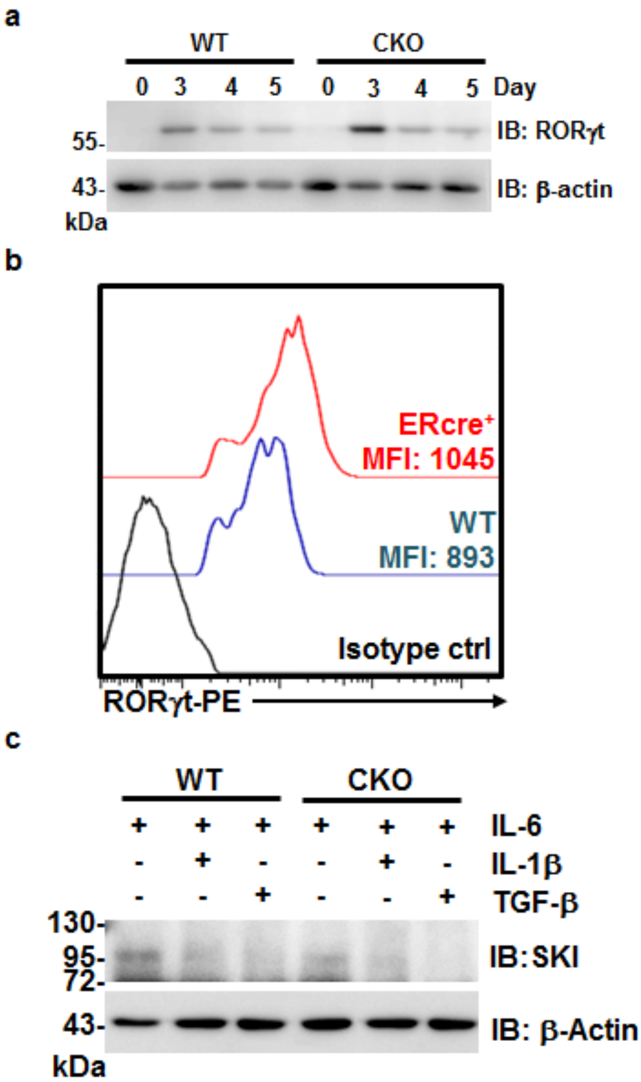

Supplemental Figure 5. The expression of RORγt is elevated in pathogenic Th17 culture in the absence of *Senp2.*

Immunoblotting (IB) showing the levels of RORγt at indicated time points of pathogenic Th17 culture derived from splenic $CD4^+$ T cells of WT and CKO mice. β-actin was used as a protein loading control. (b) The mean fluorescence intensity (MFI) of RORγt levels from FACS analysis of the $IL\text{-}17A^+CD4^+$ T cells at day 5 of 4-OHT-treated pathogenic Th17 polarizing culture derived from CKO-ER^+ and WT mice. (c) IB showing the levels of SKI on day 2 in the Th17 culture derived from splenic $CD4^+$ T cells of WT and CKO mice and treated with the indicated cytokines. β-actin was used as a protein loading control.

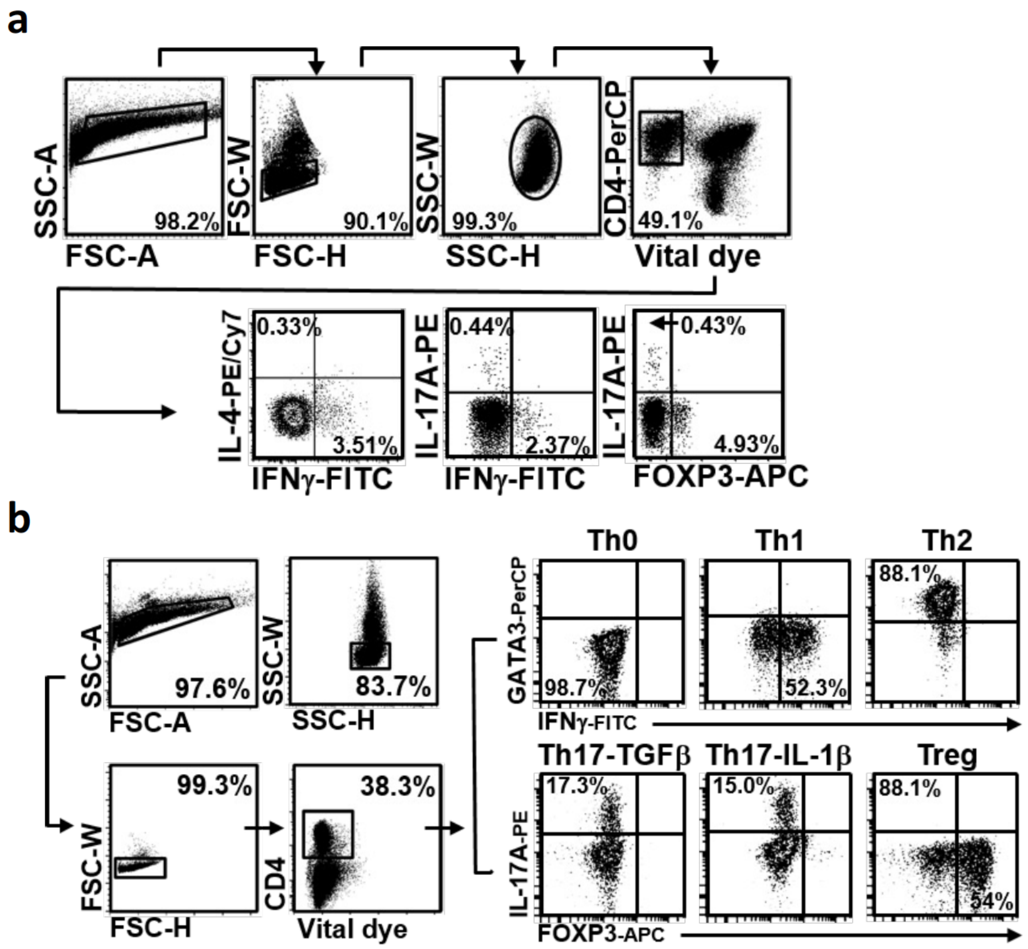

Supplementary Figure 6. Gating strategies

(a) Gating strategies for flow cytometric analysis of T cell subsets in spleen and cLN in Figure 1a and Figure S2g. (b) Gating strategies for flow cytometric analysis of T cell differentiation from naïve $CD4^+$ T cells in Figure 1d, Figure 5a, Figure 5d, and Figure S2h.

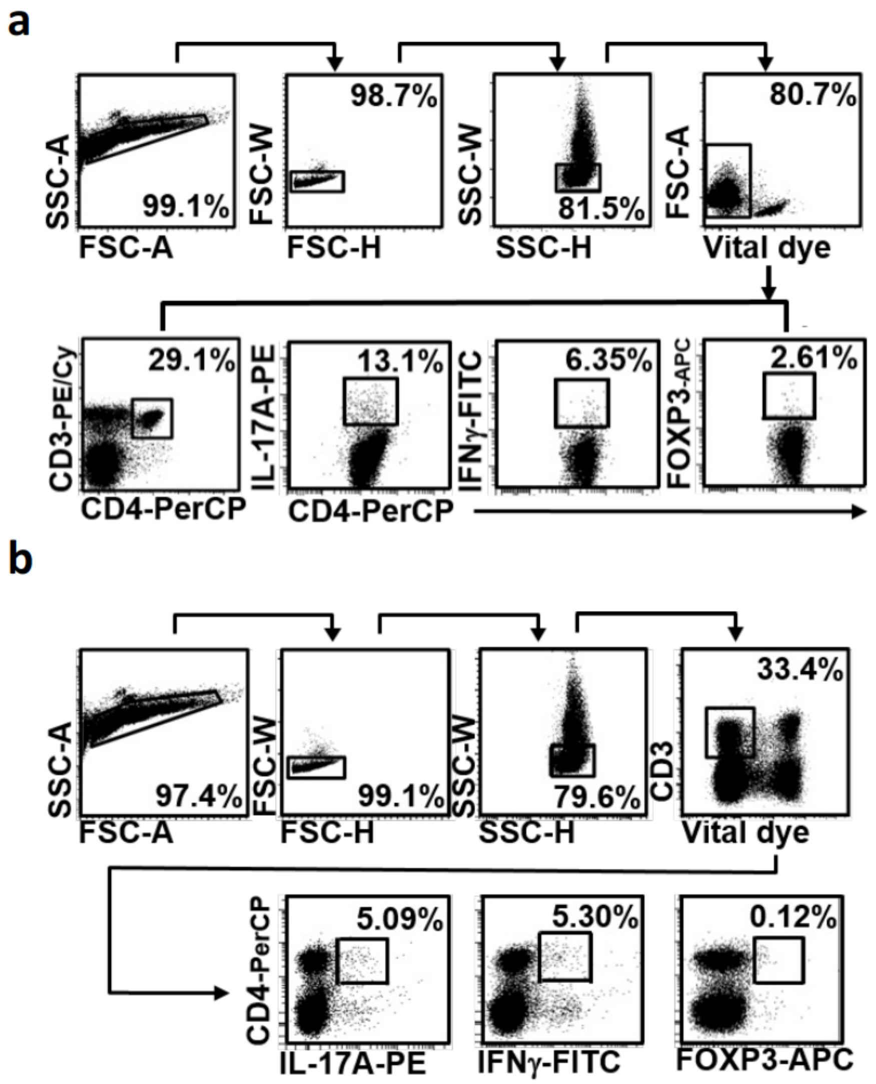

Supplementary Figure 7. Gating strategies
Gating strategies for flow cytometric analysis of T cell subsets from MLN (a) and LP (b) isolated from mouse colon in Figures 2g–n and in Figures 3f, 3m.

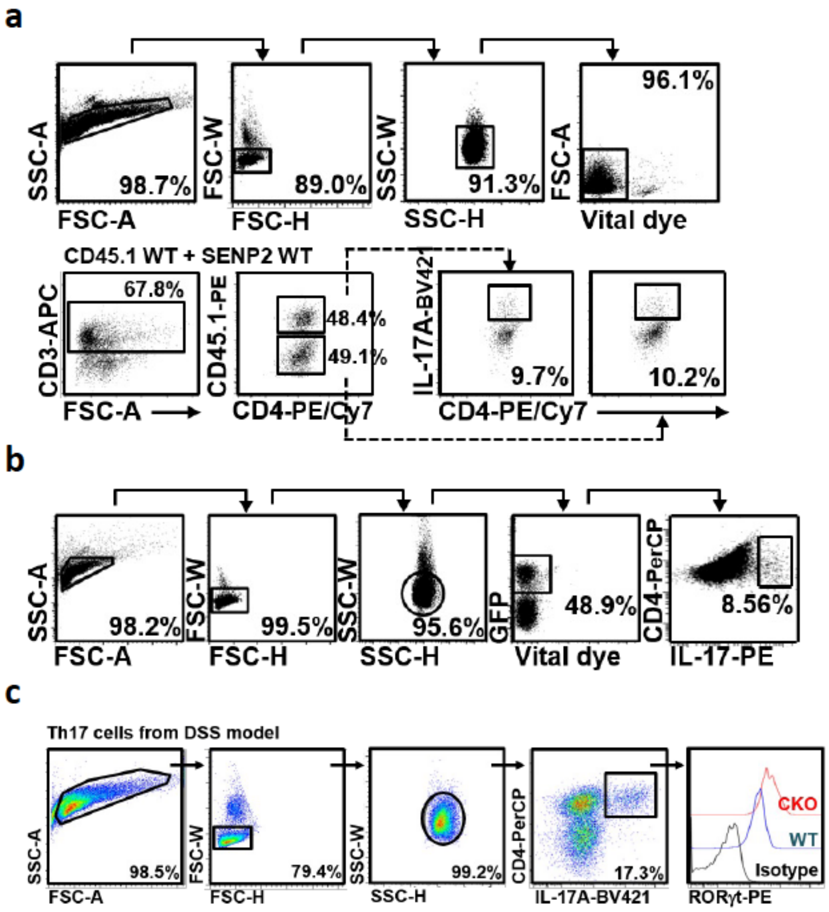

Supplementary Figure 8. Gating strategies

(a) Gating strategies for flow cytometric analysis of Th17 cells in the co-transfer experiments in Figure 3i. (b) Gating strategies for flow cytometric analysis of the frequency of IL-17A^{+}CD4^{+} cells in GFP^{+} gate in Figure 5g. (c) Gating strategies for flow cytometric analysis of the expression levels of RORγt in Figure 6i.

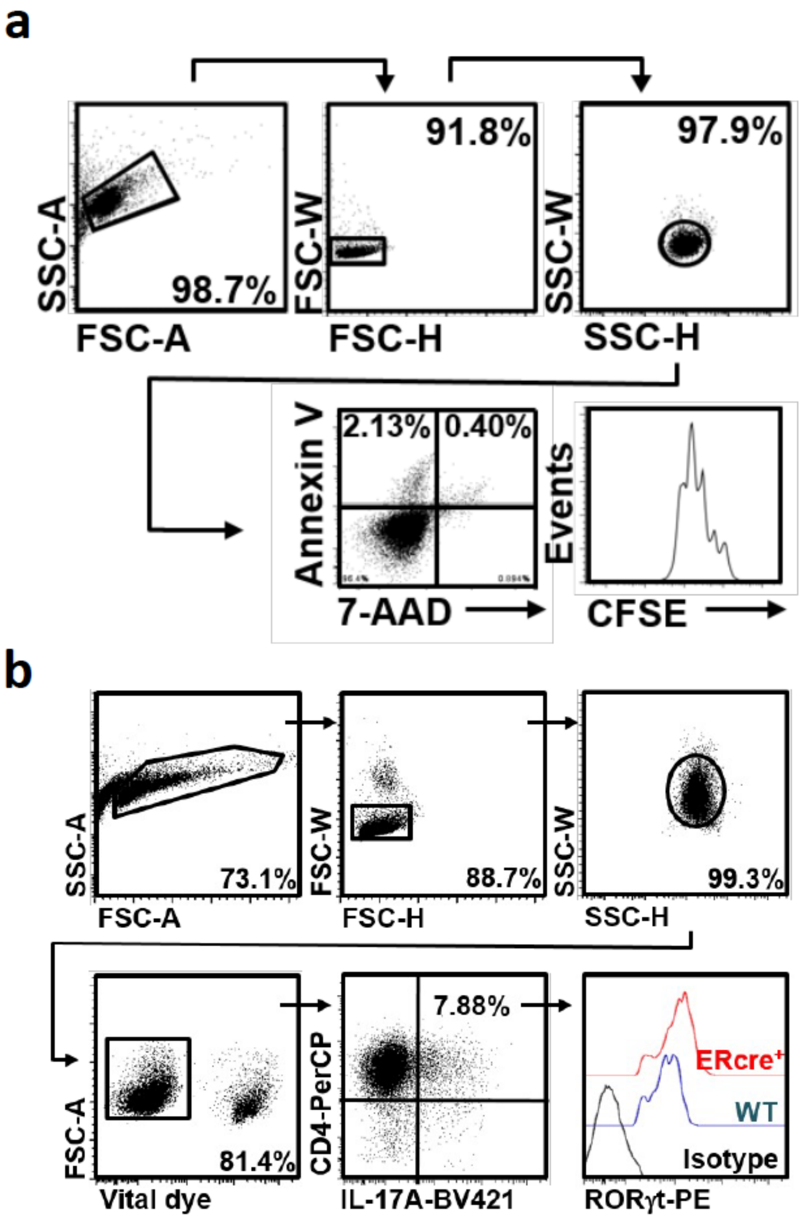

Supplementary Figure 9. Gating strategies
(a) Gating strategies for flow cytometric analysis of cell proliferation and cell apoptosis in Figure S3. (b) Gating strategies for flow cytometric analysis of the expression levels of RORγt in Figure S5b.

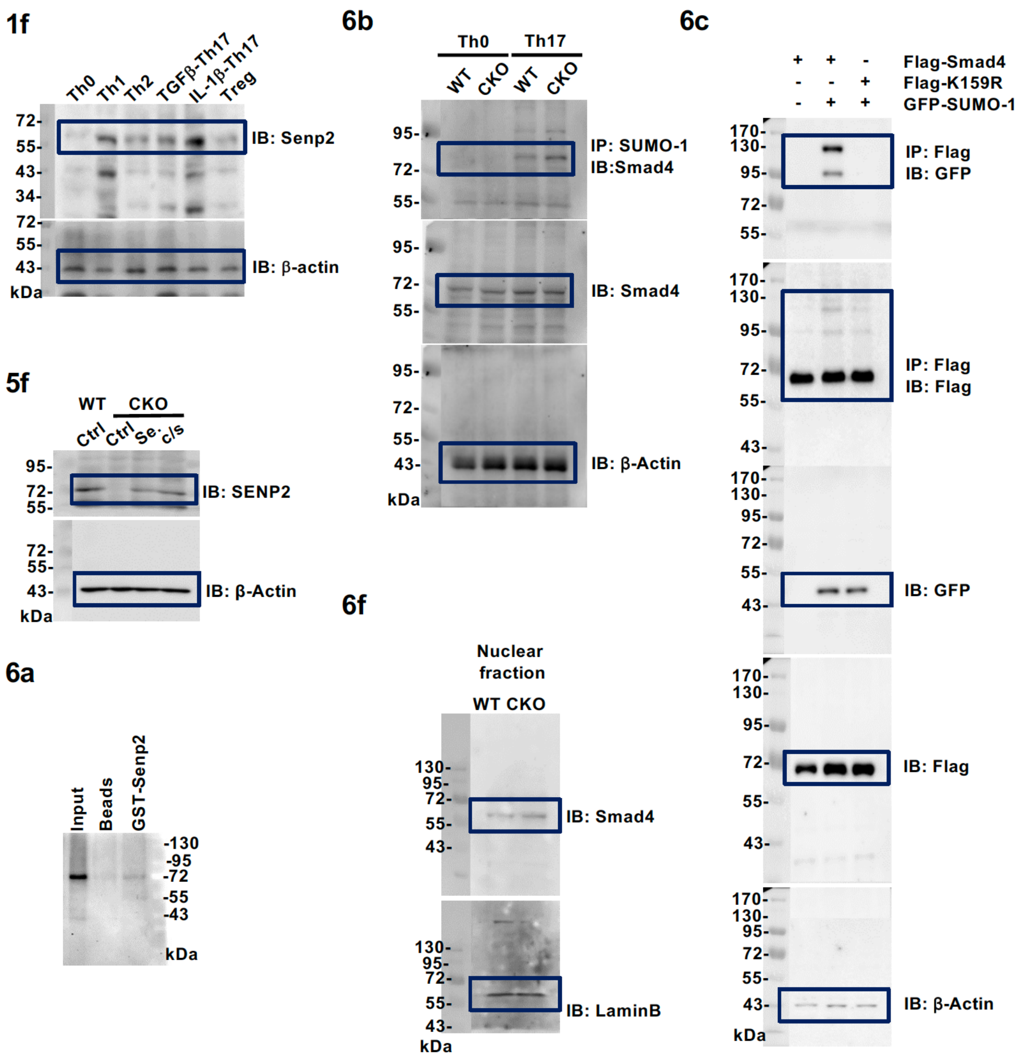

Supplementary Figure 10. Uncropped blot images

Uncropped blot images of immunoblotting analysis shown in the main figures.

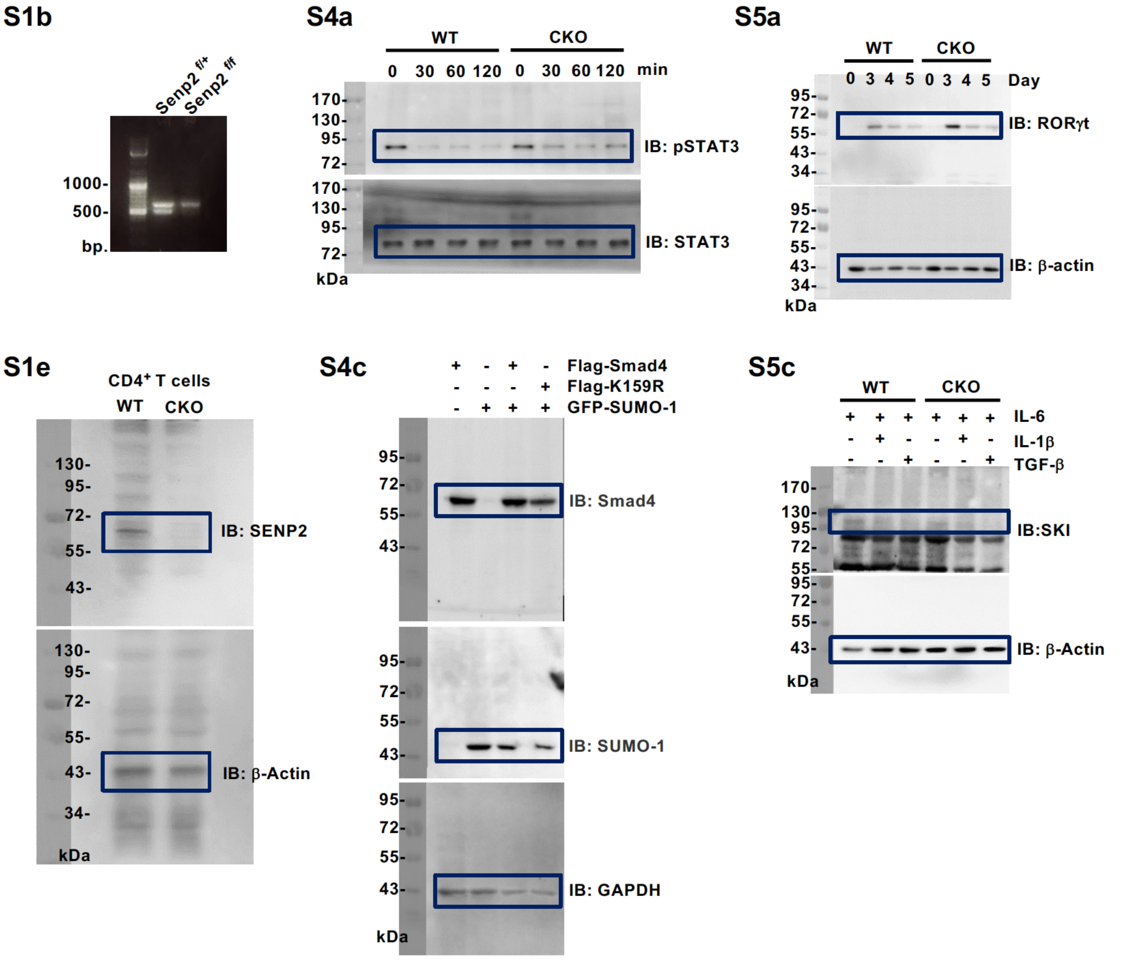

Supplementary Figure 11. Uncropped gel/blot images

Uncropped agarose gel and immunoblotting blot images shown in the supplementary figures.
