# Supplementary material for: SENP2 restrains the generation of pathogenic Th17 cells in mouse models of colitis
Source: Commun Biol. 2023 Jun 10;6:629. doi: 10.1038/s42003-023-05009-4 (PMC10257679; doi:10.1038/s42003-023-05009-4)
Supplement: Supplementary file 3 — Description of Additional Supplementary Files [file 42003_2023_5009_MOESM3_ESM.pdf]

Description of Additional Supplementary Files

File name: Supplementary Data 1

Description: This file contains source data for Figure 1 (b, c and e), Figure 2 (e, f, h, j, k, m, o, and p), Figure 3 (c, e, g, j, and m), Figure 4 (b, d, and e), Figure 5 (b, e, and h), Figure 6 (e, g, and h), Supplementary Figure 1 (c and d), and Supplementary Figure 2 (b, c, d, and f) in this manuscript.
