# Supplementary material for: SENP2 restrains the generation of pathogenic Th17 cells in mouse models of colitis
Source: Commun Biol. 2023 Jun 10;6:629. doi: 10.1038/s42003-023-05009-4 (PMC10257679; doi:10.1038/s42003-023-05009-4)
Supplement: Supplementary file 5 — Reporting Summary [file 42003_2023_5009_MOESM5_ESM.pdf]

nature portfolio

Corresponding author(s): Kuo-I Lin

Last updated by author(s): 2023/05/22

Reporting Summary

Nature Portfolio wishes to improve the reproducibility of the work that we publish. This form provides structure for consistency and transparency in reporting. For further information on Nature Portfolio policies, see our Editorial Policies and the Editorial Policy Checklist.

Statistics

For all statistical analyses, confirm that the following items are present in the figure legend, table legend, main text, or Methods section.

n/a	Confirmed	
☐	☒	The exact sample size (n) for each experimental group/condition, given as a discrete number and unit of measurement
☐	☒	A statement on whether measurements were taken from distinct samples or whether the same sample was measured repeatedly
☐	☒	The statistical test(s) used AND whether they are one- or two-sided<br>*Only common tests should be described solely by name; describe more complex techniques in the Methods section.*
☒	☐	A description of all covariates tested
☐	☒	A description of any assumptions or corrections, such as tests of normality and adjustment for multiple comparisons
☐	☒	A full description of the statistical parameters including central tendency (e.g. means) or other basic estimates (e.g. regression coefficient) AND variation (e.g. standard deviation) or associated estimates of uncertainty (e.g. confidence intervals)
☒	☐	For null hypothesis testing, the test statistic (e.g. F, t, r) with confidence intervals, effect sizes, degrees of freedom and P value noted<br>*Give P values as exact values whenever suitable.*
☒	☐	For Bayesian analysis, information on the choice of priors and Markov chain Monte Carlo settings
☒	☐	For hierarchical and complex designs, identification of the appropriate level for tests and full reporting of outcomes
☒	☐	Estimates of effect sizes (e.g. Cohen's d, Pearson's r), indicating how they were calculated

Our web collection on statistics for biologists contains articles on many of the points above.

Software and code

Policy information about availability of computer code

Data collection	Not applicable
Data analysis	Not applicable

For manuscripts utilizing custom algorithms or software that are central to the research but not yet described in published literature, software must be made available to editors and reviewers. We strongly encourage code deposition in a community repository (e.g. GitHub). See the Nature Portfolio guidelines for submitting code & software for further information.

Data

Policy information about availability of data

All manuscripts must include a data availability statement. This statement should provide the following information, where applicable:

- Accession codes, unique identifiers, or web links for publicly available datasets
- A description of any restrictions on data availability
- For clinical datasets or third party data, please ensure that the statement adheres to our policy

Raw sequencing data related to 16S rRNA genes were deposited in NCBI (BioProject accession number: PRJNA972952)

Research involving human participants, their data, or biological material

Policy information about studies with human participants or human data. See also policy information about sex, gender (identity/presentation), and sexual orientation and race, ethnicity and racism.

Reporting on sex and gender	n/a
Reporting on race, ethnicity, or other socially relevant groupings	n/a
Population characteristics	n/a
Recruitment	n/a
Ethics oversight	n/a

Note that full information on the approval of the study protocol must also be provided in the manuscript.

Field-specific reporting

Please select the one below that is the best fit for your research. If you are not sure, read the appropriate sections before making your selection.

☒ Life sciences ☐ Behavioural & social sciences ☐ Ecological, evolutionary & environmental sciences

For a reference copy of the document with all sections, see nature.com/documents/nr-reporting-summary-flat.pdf

Life sciences study design

All studies must disclose on these points even when the disclosure is negative.

Sample size	All the n numbers in each figure were provided in the Figure legends.
Data exclusions	We did not exclude any data in the manuscript.
Replication	"At least three independent biological experiments, unless otherwise indicated" was described in the Statistics and Reproducibility of Methods.
Randomization	not relevant to this study
Blinding	not relevant to this study

Reporting for specific materials, systems and methods

We require information from authors about some types of materials, experimental systems and methods used in many studies. Here, indicate whether each material, system or method listed is relevant to your study. If you are not sure if a list item applies to your research, read the appropriate section before selecting a response.

Materials & experimental systems

n/a	Involved in the study	
☐	☒	Antibodies
☐	☒	Eukaryotic cell lines
☒	☐	Palaeontology and archaeology
☐	☒	Animals and other organisms
☒	☐	Clinical data
☒	☐	Dual use research of concern
☒	☐	Plants

Methods

n/a	Involved in the study	
☒	☐	ChIP-seq
☐	☒	Flow cytometry
☒	☐	MRI-based neuroimaging

Antibodies

Antibodies used	PE/Cy7-conjugated anti-mouse CD4 (clone number: RM4-5, Cat# 100527, BioLegend), PerCP5.5-conjugated anti-mouse CD8 (clone number: 53-5.8, Cat# 980916, BioLegend), FITC-conjugated anti-mouse IFNg (clone number: XMG1.2, Cat# 505805, BioLegend), PE/Cy7-conjugated anti-mouse IL-14 (clone number: 11B11, Cat# 653811, eBioscience), PerCP5.5-conjugated anti-mouse GATA3 (clone

	number: 16E10A23, Cat# 25-7041-82, BioLegend), PE-conjugated anti-mouse GATA-3 (clone number: TWAJ, Cat# 12-9966-42, eBioscience), PE-conjugated anti-mouse IL-17 (clone number: TC11-18H10, Cat# 506903, BioLegend), BV421-conjugated anti-mouse GM-CSF (clone number: MP1-22E9, Cat# 17-7331-82, BD Biosciences), APC-conjugated anti-mouse Foxp3 antibodies (clone number: 150D, Cat# 320029, Biolegned), APC-conjugated anti-mouse CD3 (clone number: 145-2c11, Cat# 553006, BD Biosciences), FITC-conjugated anti-mouse CD3 (clone number: 145-2c11, Cat#553061, BD Biosciences), PerCP5.5-conjugated anti-mouse CD4 (clone number: RM45, Cat# 550954, BD Biosciences),PerCP5.5-conjugated anti-mouse GATA3 (clone number: 16E10A23, Cat# 653811, BioLegend),PE/Cy7-conjugated anti-mouse IL-4 (clone number: 11B11, Cat# 25-7041-82, eBioscience), PE-conjugated anti-mouse CD62L (clone number: MEL-14, Cat# 553151, BD Biosciences), APC-conjugated anti-mouse CD44 (clone number: IM7, Cat# 559250, BD Biosciences), PE/Cy7-conjugated anti-mouse CD25 (clone number:Pc61, Cat#552880, BD Biosciences). anti-RORgt (H-190, Santa cruz), anti-T-bet (H210, Santa cruz), anti-SUMO1 (D-11, Santa cruz), anti-Smad4 (D3R4N, Cell signaling), anti-STAT3-tyr705 (Cell signaling), anti-actin-HRP (Gene script), anti-Flag (Sigma), anti-GFP (Abcam), anti-SENP2 (Abcam) and anti-GAPDH (Abcam).
Validation	All the antibodies used in this study are commercially available.

Eukaryotic cell lines

Policy information about cell lines and Sex and Gender in Research

Cell line source(s)	293T, EL4 cells
Authentication	EL4 cells are purchased from ATCC
Mycoplasma contamination	293T cells were tested negative for mycoplasma contamination. EL4 cells were not tested for mycoplasma contamination.
Commonly misidentified lines (See ICLAC register)	n/a

Animals and other research organisms

Policy information about studies involving animals; ARRIVE guidelines recommended for reporting animal research, and Sex and Gender in Research

Laboratory animals	Senp2-floxed mice (Wei Hsu's lab) were bred with Lck-Cre mice (Jackson Laboratory) in C57BL/6 background. Senp2-floxed mice were bred with R26-ERCre mice (Jackson Laboratory) or RORct-Cre mice (Jackson Laboratory) in C57BL/6 background. CD45.1 mice (Jackson Laboratory) were in C57BL/6 background.
Wild animals	n/a
Reporting on sex	We used both genders of mice for experiments. No obvious differences were found in the results.
Field-collected samples	This study did not involve samples from the field.
Ethics oversight	The experimental protocol of animal study was approved by Academia Sinica's Institutional Animal Care and Utilization Committee.

Note that full information on the approval of the study protocol must also be provided in the manuscript.

Flow Cytometry

Plots

Confirm that:

☒ The axis labels state the marker and fluorochrome used (e.g. CD4-FITC).

☒ The axis scales are clearly visible. Include numbers along axes only for bottom left plot of group (a 'group' is an analysis of identical markers).

☒ All plots are contour plots with outliers or pseudocolor plots.

☒ A numerical value for number of cells or percentage (with statistics) is provided.

Methodology

Sample preparation	Splenic CD4 T cells were stimulated with PMA (50 ng/ml) and ionomycin (1 mg/ml) (Sigma-Aldrich) in the presence of monensin (eBioscience) for 5 h, and then stained with CD4 antibodies. After fixation and permeabilization by eBioscience™ Foxp3 / Transcription Factor Staining Buffer Set (Invitrogen™), cells were stained with intracellular antibodies for 30 min at 4° C, and washed with PBS for 3 times, followed by FACS analysis with flow cytometry FACScanto.
Instrument	BD FACScanto
Software	FACScanto and FlowJo
Cell population abundance	The DN thymocytes divided into DN1 (CD25-CD44+), DN2 (CD25+CD44+), DN3 (CD25+CD44-) and DN4 (CD25-CD44-). To

Cell population abundance	identify Th subsets, CD4+IFNg+ Th1 cells, CD4+GATA3+ Th2 cells, CD4+IL-17A+ Th17 cells, GM-CSF+IL-17+CD4+ pathogenic Th17 cells and CD4+Foxp3+ Treg cells.
Gating strategy	We used FSC-A and SSC-A to identify all cells, and then used FSC-A and FSC-H to gate for single cells.

☒ Tick this box to confirm that a figure exemplifying the gating strategy is provided in the Supplementary Information.
